# The Greely Expedition and Anthropophagy

**Published:** 1884-10-20

**Authors:** 


### THE GREELY EXPEDITION AND ANTHROPOPHAGY.

The story of the sufferings of the members of the Greely Arctic expedition is now known to the world, and shows a depth of privation that is astounding even to those familiar with expeditions to the frozen zone. The tale of woe is, it is hoped, ended with the seemingly undeniable proof that the survivors were compelled to sustain life by feeding on the remains of their unfortunate comrades, who could not resist the combined foes of extreme cold and hunger.

The Medical Record has had a private interview with Lieutenant Greely, and publishes some interesting observations on the length of time an inhabitant of the temperate zone can live in the Arctic regions. Lieutenant Greely ascertained that such people can live in the land of winter for a number of years if they be in good physical condition, but found that there was a marked limit to their powers of endurance. For a long time the system seems able to withstand the rigors of the climate, but a decided decrease in nervous and muscular energy, and in the calorigenous process, is noticed at the end of the first year. The second winter was found harder than the first, the favorable point being that the experience of the previous winter enabled them to better husband their resources. The third winter was still harder to bear: there was a loss of muscular tone, less vital reaction, and rheumatisms were frequent. Lieutenant Greely doubted if his men could have lived five years in the climate, even with the best that could be provided in the way of food and shelter. He thinks they would, however, be alive to-day had the supply of food been maintained. The party found that the breathing of such cold air quickened the heart's action, and the respiratory, assimilative and digestive processes. It seemed to have something of the effect of breathing pure oxygen. No such thing as pulmonary troubles were known; nor were any of the party even bothered with catarrh.

So much for Lieutenant Greely's observations. They speak for themselves and it is easy for the intelligent reader to draw his own deductions.

The point that chiefly interests the public, professional and lay, is that of cannibalism: was, or was it not resorted to, and if so, was it excusable on the part of the survivors? There can be little doubt in the mind of any one who has followed the daily newspaper reports of the details relating to this part of the affair that the flesh of the dead was, toward the last, the sole sustenance of the few who lived to return to their homes. The ravings of the half-crazed men when first rescued, the partial stories of sailors of the relief ships, and the revelations made at the post mortem examination of Lieutenant Kislingbury and others, would establish the fact in the mind of any one. A conclusive point was the finding of epidermis in the stomachs of some of the dead. While a mistake might be made, and easily, in attempting to decide whether a specimen of muscular tissue was or was not human, it would not be so difficult to tell that a piece of skin belonged to a man. There can, then, be no doubt that some of the rescued members of the Greely expedition practiced anthropophagy.

Were they justified in so doing? It is very easy for a man who sits in his parlor after a hearty dinner, smoking a soothing cigar or pipe, to shudder when the question is asked, and say under no circumstances would a human being be justified in feeding on the flesh of one of his kind; but the question would present itself in another aspect to the man who had spent nearly three years in a dreary and desolate land of ice and snow; who had been for months without the food proper to sustain the vital energies so heavily drawn upon; who was feeling the pangs of slow starvation and saw a miserable death staring him in the face; who felt life was dear in what the future might hold if he should be rescued. To such a man, desperate and with blunted intellect, the matter of keeping alive by eating the flesh of the dead companions, who are not injured by the act, do s not, can not seem so horrible. Let any man imagine himself in such straits as were these few survivors of the expedition, and say that he, under similar circumstances, would not have taken the same means of fighting death that these men did.

The natural rebellion of the feelings against the suggestion of anthropophagy is calculated to drive a scientific estimate of the nutritive value of human flesh quite out of the question in any consideration of the subject which the horrors of the Greely expedition may incite. And yet the unspeakable sufferings of the party and the straits to which they were driven but tend to force the scientific features of the case, divested of all sentimentality, upon the attention of the medical profession particularly. What has occurred may occur again, and it may be necessary for men to eat the dead of their kind again to preserve themselves from a miserable death. In such a case it would be unfortunate, to say the least, should false notions of the food-property of such flesh deter them from resorting to it in the hour of their dire extremity. It is popularly supposed, and the belief is perhaps not confined to the laity, that human flesh is inherently poisonous in its nature. The experience of the Greely party, however, sustains the only conclusions deducible from physiologial investigations of this matter, viz: that this popular belief is erroneous. The secretions of the human animal are not so essentially different in their nature from those of inferior animals as to preclude experiments on the latter from being made the basis of even therapeutical deductions in the case of man. The milk of the woman differs only from that of the cow in the matter of a constituent which adapts it more fully to the digestive capacity of the child, and there is no sufficient reason for the assumption that human flesh differs any more in its properties from that of inferior animals than that its secretions are essentially different. It is nitrogenous food of the most perfect order, and, probably, could it be obtained under as favorable conditions as obtains in the case of the flesh which is sold in the shambles its taste would in no respect be different from that of the latter.

It is unfortunate that men should, either through their own voluntary foolhardy undertakings or through the vicissitudes of legitimate enterprise, be reduced to a condition of starvation; but, this condition having been reached, the question of eating the flesh of their kind becomes an ethical, and not a scientific one.—*Medical Age.*

